# Feeder Cells at the Interface of Natural Killer Cell Activation, Expansion and Gene Editing

**DOI:** 10.3389/fimmu.2022.802906

**Published:** 2022-02-11

**Authors:** Mark Gurney, Soumyadipta Kundu, Shubham Pandey, Michael O’Dwyer

**Affiliations:** ^1^ Department: Apoptosis Research Centre, National University of Ireland Galway, Galway, Ireland; ^2^ ONK Therapeutics, Galway, Ireland

**Keywords:** NK cells, expansion, transposon, genome editing, immunotherapy

## Abstract

Genome engineered natural killer (NK) cell therapies are emerging as a promising cancer immunotherapy platform with potential advantages and remaining uncertainties. Feeder cells induce activation and proliferation of NK cells *via* cell surface receptor-ligand interactions, supported by cytokines. Feeder cell expanded NK cell products have supported several NK cell adoptive transfer clinical trials over the past decade. Genome engineered NK cell therapies, including CAR-NK cells, seek to combine innate and alloreactive NK cell anti-tumor activity with antigen specific targeting or additional modifications aimed at improving NK cell persistence, homing or effector function. The profound activating and expansion stimulus provided by feeder cells is integral to current applications of clinical-scale genome engineering approaches in donor-derived, primary NK cells. Herein we explore the complex interactions that exist between feeder cells and both viral and emerging non-viral genome editing technologies in NK cell engineering. We focus on two established clinical-grade feeder systems; Epstein-Barr virus transformed lymphoblastoid cell lines and genetically engineered K562.mbIL21.4-1BBL feeder cells.

## Introduction

Natural killer (NK) cell-based adoptive cell transfer (ACT) is a promising experimental approach to cancer immunotherapy. The ability to administer NK cells across HLA barriers, without a risk of graft-versus-host disease has enabled NK cell products from a variety of cell sources to be evaluated in clinical trials (1–4). The therapeutic potential of unmodified NK ACT has been most apparent to date in the setting of hematological malignancy (1, 5, 6). The traditional paradigm that NK cells are more challenging to genetically engineer relative to T-cells is evolving (7, 8). Clinical scale gene editing of NK cells is established, most prominently through the addition of a chimeric antigen receptor (CAR) (3). CAR-NK cells add a potent layer of antigen specific activation to a complement of NK cell activating and inhibitory receptors which are the basis of innate and alloreactive NK cell recognition.

**Figure 1:**
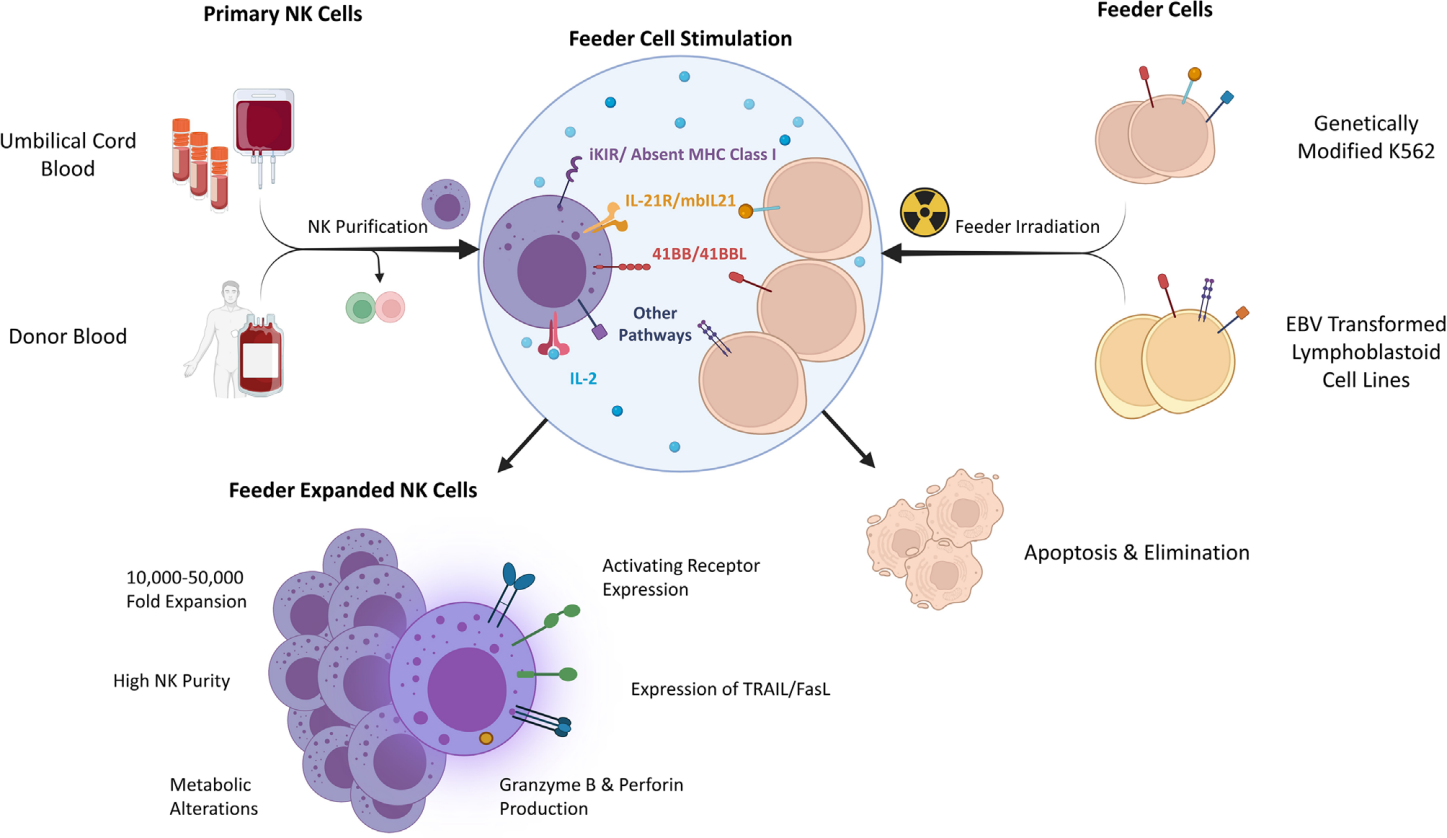
Determinants and characteristics of feeder expanded NK cells. iKIR – inhibitory killer immunoglobulin receptor, EBV, Epstein-Barr Virus; mbIL21, membrane bound IL-21; TRAIL, TNF-related apoptosis-inducing ligand.

Primary NK cells are sourced from donor peripheral blood (PB) or umbilical cord blood (UCB). Although both sources are the basis of investigational therapies, UCB contains additional NK cell progenitors and is collected non-invasively or from existing banked material (9–11). Large scale NK cell production from small initial quantities relies upon feeder cells which naturally, or *via* further engineering, present ligands for NK cell receptors driving profound *ex vivo* NK cell expansion when combined with cytokines (**Figure 1**) (12, 13). To avoid a risk of proliferation and contamination, feeder cell lines are γ-irradiated prior to use. While the NK cell yield varies between donors, several approaches have been used to support clinical trials (6, 14, 15).

Feeder cells are integral to clinical scale, genetically modified primary NK cell production protocols (3, 16–18). This contrasts with genome engineered T-cells where antibody conjugated beads are the typical activating stimulus, induced pluripotent stem cell (iPSC) derived NK cells (a distinct and promising domain of investigational NK therapies) which are genetically engineered prior to differentiation and expansion, and NK cell lines (e.g. NK-92) with autonomous growth potential (2, 4, 17). The role of feeder cells in generating genetically modified primary NK cell products is multifaceted. Initial feeder cell stimulation may enhance the efficiency of gene editing through simple induction of cell division or more complex effects reflecting altered gene expression. Repeated feeder cell stimulation can support cellular recovery and expansion to clinical scale despite extensive *ex vivo* manipulation. This central role for feeder cells in the era of genome engineering compels us to examine the relationship between these vital tools. Herein we consider the interface between feeder cell-based primary NK cell activation and expansion with gene editing, focusing on prominent clinical-grade feeder cell approaches, Epstein-Barr Virus transformed lymphoblastoid cell lines (EBV-LCL) and genetically engineered K562.mbIL21.4-1BBL cells.

## Prominent Feeder Cells for NK Expansion

*In vitro* infection of a mixed lymphocyte population with EBV creates an immortalized cell line with the characteristics of proliferating B-cells despite expression of relatively few viral genes (19). Early reports of preferential expansion of NK cells which occurs after co-culture of EBV-LCLs with PB mononuclear cells recognized the importance of both IL-2 and cell contact (20, 21). EBV-LCLs naturally express 4-1BBL (CD137L), the ligand for 4-1BB- an inducible stimulatory receptor on activated NK cells which is also upregulated by interactions with the Fc portion of antibodies (22, 23). Stimulation through 4-1BB is an important contributor to clinical scale NK cell expansion. EBV-LCLs express other ligands relevant to NK cell activation and expansion, including CD155, CD48 and CD58, through interactions with the NK cell activating receptors DNAM-1, 2B4 and CD2 respectively (24, 25). Dr. Richard Childs and colleagues at the National Institutes of Health (NIH) pioneered the use of EBV-LCL feeder cells as a clinical-grade NK cell expansion technique (26). Using enriched NK cell populations, mean 1,000-2,000-fold expansions are observed over 14 days and this process was subsequently automated using the GMP-compliant Miltenyi CliniMACS Prodigy (12, 27). Robust expansion of UCB NK cells has also been described from small amounts of starting material (28). The addition of a single IL-21 exposure at the outset enhances NK cell proliferation and enables prolonged expansion, overcoming senescence (29). The EBV-LCL expansion process was used to support a clinical trial at the NIH of expanded NK cells combined with Bortezomib, a sensitizing agent to the death ligand TRAIL, across a range of malignancies (NCT00720785).

Initial descriptions of the immunophenotype and *in vitro* function of EBV-LCL expanded NK cells, have been complemented by gene expression profiling (GEP) (12, 27, 30). Relative to freshly isolated NK cells, expanded populations adopt an activated phenotype with increased expression of activating receptors NKG2D, NKG2C, NKp30, NKp44, DNAM-1 and key effector molecules TRAIL, FasL and Granzymes. Described alterations in inhibitory receptors include increases in NKG2A positive cells and relative increases in both KIR2DL2/3 and TIGIT (12, 27). The diverse effects that this expansion process has on NK cells is highlighted by GEP with prominent upregulation of genes involved in metabolism, cytotoxicity and cellular growth (30). Many of these beneficial characteristics appear to be largely dependent on continued IL-2 exposure, and exogenous IL-2 administration alone may not overcome a loss of activation characteristics encountered *in vivo* (29).

An alternative, prominent feeder cell approach to NK cell expansion began with the recognition that K562, an erythroleukemia cell line deficient in HLA class I and a common target cell for *in vitro* NK cell cytotoxicity assays, provides a contact dependent expansion stimulus to NK cells (31). Expression of additional NK cell stimulatory molecules, primarily 4-1BBL and membrane bound (mb) cytokines (IL-15 or IL-21), introduced by viral transduction or DNA transposons, greatly enhances the expansion potential of K562 (32, 33). The impact of K562.4-1BBL feeder cell expression of mbIL15 or mbIL21 on the characteristics of expanded NK cells was reported by Denman et al. (13). Feeder cell expression of mbIL15 produced a mean 825-fold NK expansion (day 21), with telomere shortening relative to non-expanded NK cells and senescence characterized by loss of proliferation between weeks 4 and 6. Feeder cell expression of mbIL21 produced a mean 47,697-fold NK cell expansion (day 21), with an increased telomere length compared to baseline and sustained NK cell proliferation to week 6. Feeder cell expression of either mb cytokine was associated with a similar NK cell phenotype, including high expression of natural cytotoxicity receptors (NKp30, NKp44, NKp46) and KIR. Prominent activation and proliferation characteristics including high levels of granzyme B and perforin were observed by GEP. Relative to mbIL15, feeder cell expression of mbIL21 produced expanded NK cells with a similar *in vitro* cytotoxicity, but greater cytokine production. Feeder cell expression of mbIL21 has been shown to enhance NK cell metabolism through STAT3/cMyc pathway signaling (34). Several groups have reported on the use of K562.mbIL21.4-1BBL feeder cells to expand NK cells used in early phase clinical studies across PB and UCB NK cells, including the most prominent example of a CAR-NK cell therapy reported to date (3, 6, 14, 35). Membrane particles derived from K562.mbIL21.4-1BBL feeder cells have also been extracted and applied to NK expansion, overcoming the risk of viable feeder cell contamination, despite irradiation (36).

**Figure 2:**
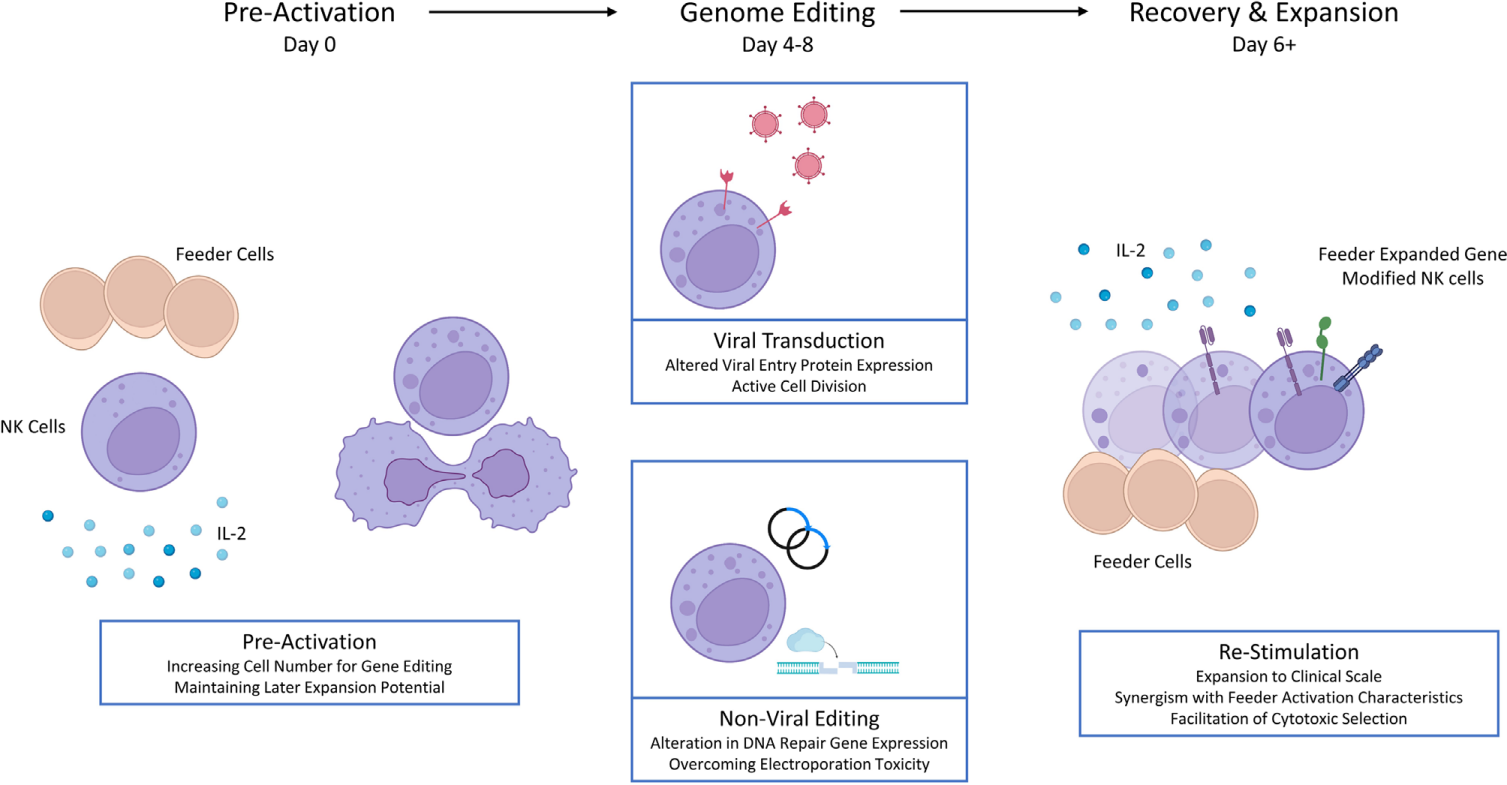
Feeder cell stimulation and primary NK cell genome editing.

The characteristics of these prominent feeder cell approaches are summarized and compared in **Table 1**. Both are being exploited to facilitate clinical-scale primary NK cell genome editing, with important benefits of feeder cell stimulation highlighted in **Figure 2**. The reliance upon feeder cell expansion in current gene editing protocols has implications for the characteristics of these products. Some features may be synergistic with specific gene edits such as the generation of CAR-NK cells. For example, death ligands TRAIL and FasL are implicated in supporting NK cell serial killing, and subsequent secretion and/or shedding of TRAIL may trigger apoptosis of target antigen negative populations as described for CAR-T cells (45, 46). Alternatively, some characteristics are less desirable, such as the loss of activation features that may occur *in vivo*. Here, rational genome editing targets may overcome these limitations. Introduction of an IL-15 gene allows for autonomous production and enhances the functionality and persistence of CAR-NK cells, without inducing systemic alterations in IL-15 (42). Knockout of cytokine-inducible SH2-containing protein (*CISH*), a negative regulator of IL-15 signaling, has multiple effects relevant to NK cell therapies including enhanced cytotoxicity, metabolism and persistence which have been characterized in pre-clinical *in vivo* models (47–49). The induction of memory like characteristics, by transient exposure to IL-12, IL-15 and IL-18 *in vitro*, offers an alternative approach to enhancing *in vivo* NK cell performance. The beneficial characteristics of these cytokine induced memory like (CIML) NK cells (including enhanced *in vivo* persistence) and CAR triggered activation are synergistic, however the interaction between CIML-NK features and feeder mediated expansion is less clear (50).

**Table 1:** Comparing two common feeder cells for NK expansion.

Feeder Cells	EBV-Lymphoblastoid Cell Lines	mbIL21-4-1BBL expressing K562
**Source**	Infection and transformation of B-lymphocytes with EBV *in vitro*	Viral/non-viral transduction of parental K562 erythroleukemia cell line.
**Ligand (receptor) candidates favoring NK expansion**	*Soluble Cytokines:* rhIL-2 ± IL-21 *Primary Candidates:* 4-1BB Ligand (4-1BB), CD48 (2B4) (24, 29)	*Soluble Cytokines:* rhIL-2 *Primary Candidates:* Low HLA-A and HLA-B expression (iKIR), 4-1BB Ligand (4-1BB), mbIL-21 (IL-21R) (13)
	*Additional Candidates:* CD58 (CD2), OX40L (OX40), CD155 (DNAM-1) (25, 37, 38)	*Additional Candidates:* NKG2D ligands (NKG2D), natural cytotoxicity receptor ligands (NKp30, NKp44, NKp46) (39)
**Selected Fold Expansion**	Purified PB NK cell, 2-weeks: 815-3267 fold (12), 2900-fold (with IL-21) (29)	Purified PB NK cells, 2-weeks: ~2,000-fold (13)
**Characteristics of Expanded NK Cells**	Increased Receptor Expression: NKG2D, NKG2C, NKp30, NKp44, DNAM-1, NKG2A, KIR2DL2/3, TIGIT. Effector Molecule Production: TRAIL, FasL, Granzymes	Increased Receptor Expression: NKG2D, NKp30, NKp44, NKp46, DNAM-1, CD16, NKG2A Effector Molecule Production: Granzyme B, Perforin
**Examples of Genome Editing Combinations**	Lentiviral Transduction (40) TcBuster DNA Transposon (41)	Retroviral Transduction (3, 42) TcBuster DNA Transposon (43)
**Clinical Trial Examples**	NCT00720785 (multiple malignancies, with Bortezomib)	NCT01904136 (haploidental donor blood, AML) (44), NCT03056339 (CD19 CAR-IL-5-iCas9 NK cells, cord blood, CD19+ malignancies) (3), NCT01729091 (cord blood, multiple myeloma)

## Feeder Cells and Viral Transduction

Retroviral vectors are established gene delivery tools based upon the principle of reverse transcription of a viral RNA genome into double stranded DNA, which is then integrated into the host cell genome by the enzyme integrase, allowing for stable transgene expression (51). Gamma retroviruses efficiently deliver genes to replicating cells *via* nuclear membrane breakdown during mitosis, while lentiviral vectors can also transduce resting cells. Each of these classes are successfully used in manufacturing regulatory approved CAR-T products (52, 53). Several groups have described the role of K562.mbIL21.4-1BBL feeder cells in facilitating NK cell viral transduction. Using an RD114 pseudotyped retroviral vector Streltsova et al. described the transduction of feeder activated but not resting NK cells. Interestingly, the small, mature CD57^+^ NK cell subset detectable within the expanded population, failed to proliferate in response to the feeder stimulus and remained resistant to transduction, emphasizing the role of cellular division (54). In another example where feeder-free expansion and K562.mbIL21.4-1BBL feeder cells were compared, both facilitated transduction with a baboon envelope pseudotyped lentiviral vector, however subsequent re-expansion of sorted populations relied upon feeder cell stimulation (55). Interestingly, one proposed mechanism for the enhanced transduction achieved after activation relative to freshly isolated NK cells was upregulation of the viral entry proteins ASCT1 and ASCT2. Given the importance of cellular replication to successful retroviral engineering and the clinical-scale expansion required, the robust activating and expansion stimulus delivered by feeder cells has been central to the clinical translation of retrovirally engineered CAR-NK cells. In their phase I clinical trial report, Liu et al. describe an UCB derived, expanded NK cell product which expresses a CD19 CAR, IL-15, and an inducible caspase 9 suicide gene. This multi-cistronic gene was introduced on day 6 using a retroviral vector, after initial stimulation of purified UCB NK cells by K562.mbIL21.4-1BBL feeder cells. Later restimulation allowed for expansion sufficient to provide multiple clinical doses (3, 42). This groundbreaking study, which demonstrated clinical responses in the context of B-cell malignancies and long-term persistence of CAR-NK cells, established the promise of the CAR-NK cell platform.

Recently, Allan et al. reported on the optimization of lentiviral transduction in primary NK cells which further explores the role that feeder cells play in the delicate balance of gene editing and expansion potential (40). Notably, while initial activation with IL-2 alone allowed for successful transduction relative to unstimulated NK cells, subsequent EBV-LCL feeder cell stimulated expansion was limited. Incorporation of EBV-LCL pre-stimulation modestly reduced integration rates but provided an overall higher yield of transduced cells at later timepoints reflecting a maintained expansion potential. The group went on to demonstrate that 5-7 days pre-activation prior to lentiviral transduction was optimal, balancing efficiency and fold-expansion, while the purity of transduced cells could be enhanced by immunomagnetic selection based on truncated protein selection markers. NK cells rapidly overwhelm dissipating feeder cells and were harvested for genetic engineering without repeated selection. In experiments where direct comparison was made between EBV-LCL and K562.mbIL21.4-1BBL feeder cells, similar results were obtained for transduction efficiency and expansion capacity. A distinct lymphoblastoid cell line, 721.221, engineered to express mb IL-21, has also been successfully combined with retroviral transduction to create CAR-NK cell products in a recent pre-clinical report (56).

Viral transduction approaches generally confer less toxicity to immune cells relative to electroporation with DNA based cargo. This improved viability, could be the basis of successful integration of feeder-free expansion approaches in the future. Feeder-free NK cell expansion relies upon cytokines and stimulating supplements, or antibodies, but in general results in a lower fold expansion. Feeder-free approaches offer logistical benefits and overcome the risk of viable feeder cell contamination, despite irradiation. Clinical scale examples of feeder-free expansion from primary NK cells have been reported, including using the supplement nicotinamide which confers a multitude of desirable characteristics to the expanded NK cell product beyond increasing cell number, including enhanced cytotoxicity and *in vivo* persistence (57, 58). Feeder-free approaches tend to rely upon larger volume apheresis product as a starting material, and to our knowledge have not been successfully combined with clinical-scale, stable gene editing of primary NK cells to date (59).

## Feeder Cells and Non-Viral Gene Editing

Non-viral gene editing approaches are increasingly being applied to NK cells successfully and this field has been recently reviewed (8). Electroporation remains the most widely investigated non-viral approach to deliver diverse cargo including mRNA, DNA and protein to NK cells. This allows for transient gene expression from a non-integrated DNA plasmid or mRNA, or, genome level modification and stable expression when combined with transposon technology, or knock-in after delivery of endonucleases and a DNA template (8). Non-viral genome editing has potential safety advantages relative to viral transduction, but lacks the longer term safety data that is available for clinical products manufactured using viral approaches (60). DNA transposons are versatile gene vector systems, involving co-delivery of a transposon plasmid containing the genetic cargo, and a transposase enzyme which cuts and pastes the transposon into genomic DNA by recognition of flanking inverted terminal repeat (ITR) sequences (61). A variety of factors influence the efficiency of NK cell electroporation, including the type and concentration of cargo, pulse characteristics and electroporation buffer (62). The ultimate rate of stable integration achieved after DNA transposon electroporation may also be impacted by differences in the recovery and expansion rate of modified and non-modified populations, reflecting recovery conditions and the timing of restimulation.

Feeder cell-based activation and expansion is integral to early reports of the DNA transposon-based gene editing of primary NK cells. K562.mbIL21.4-1BBL feeder cell pre-activation supports enhanced electroporation efficiency and recovery when delivering CRISPR/Cas9 cargo (63). In a recent pre-print, Pomeroy et al. describe the application of the TcBuster™ transposon system to primary NK cells using the K562.mbIL21.4-1BBL pre-activation approach (43). Day 4 is identified as the optimal time for transposon delivery by electroporation, which may reflect the later upregulation of DNA sensors, and transposition efficiency was further enhanced by ribonuclease inhibitor pre-treatment of NK cells (transposase enzyme mRNA was simultaneously delivered with the DNA transposon). Their approach also incorporates Nanoplasmid™ to reduce the size of the plasmid selection cassette aiming to enhance DNA delivery, while others have applied minicircle technology with a similar goal (64, 65). Repeat stimulation 48h post electroporation allowed for clinically relevant expansion characteristics. Our group and collaborators have also recently applied TcBuster™ to primary NK cells using EBV-LCL pre-activation and expansion to successfully generate CAR-NK cells (41).

Viral RNA size may be a limiting factor for the efficiency of transduction in primary NK cells (40). DNA transposons allow for the delivery of larger transgenes and potentially can reduce the cost and variability of immune cell engineering. This ability has been leveraged to deliver multi-cistronic genes including a cytotoxic selection marker, allowing production of a homogenous gene modified product (66). Using these selection systems combined with robust feeder driven expansion would facilitate the outgrowth of gene modified cells from a lower percentage of transposition – an important consideration, as the ideal balance of efficiency, cellular toxicity, and subsequent expansion capability remains unclear. This was highlighted by the incidence of product derived lymphomas in two patients treated with a *Piggybac* transposon-based CAR-T product which were recently reported, although the transposon system was not directly implicated in this occurrence, despite extensive investigation (67).

The combination of endonucleases, with homologous DNA templates has been successfully applied to stable gene editing, including in NK cells. Cas9 mediated double strand breaks, repaired by homologous recombination or non-homologous end joining, can lead to integration of a tailored DNA template. This allows for targeted gene insertions to genetic safe-harbour sites, and simultaneous gene knock-out. The most effective examples of this approach to date, rely upon a non-integrating adenoviral vector to deliver the DNA template, shortly after electroporation delivery of gRNA/Cas9 complexes (68, 69). Considerably greater integration was achieved using a self-complementary adenovirus vector, providing for rapid availability of the dsDNA template for DNA repair. Notably, NK cell expansion with K562.mbIL21.4-1BBL feeder cells was implicated in the optimisation of this approach. Kararoudi et al. demonstrated upregulation in genes involved in homologous recombination (BRCA1 and BRCA2) in expanded NK cells, likely contributing to an improved efficiency of knock-in. Recently, efficient gene knock-out and knock-in of NK cells maintained in a feeder-free media was described using a highly active mutant of AsCas12a, a promising future approach to non-viral gene editing (70).

## Discussion

The landscape of investigational NK cell therapies has rapidly diversified. iPSC-NK, and donor-derived, primary NK cell products may become effective, off-the-shelf immunotherapies. Genome editing, supported by novel technologies and adaptation of existing techniques to NK cells appears fundamental to achieving this potential. Current protocols for clinical-scale genome editing of primary, donor-derived NK cells rely upon feeder cell stimulation. Initial stimulation increases the NK cell quantity for gene editing and improves the efficiency of retroviral transduction and transposon delivery. Repeat stimulation supports NK cell recovery and subsequent expansion, allowing for clinical scale and multi-dose manufacture. The characteristics of primary NK cells expanded with each of the feeder cell systems discussed herein apply to the final product. This may be beneficial by protecting against senescence during expansion or create a barrier through potential loss of activation characteristics occurring *in vivo.* Although few reports directly compare the two prominent feeder approaches discussed here, they share core activating pathways, and both are being successfully applied to support NK cell genome editing. Proposed feeder-free and novel approaches to NK expansion should also be evaluated for their performance in this critical supportive role to NK cell genome editing (36, 71, 72).

## Author Contributions

MO’D conceived of the review and contributed to content and editing. MG wrote the manuscript and compiled figures. SK contributed to literature review and editing. SP contributed to literature review and editing. All authors contributed to the article and approved the submitted version.

## Funding

Author MG is supported by Irish Clinical Academic Training (ICAT) Program fellowship funding. ICAT is supported by the Wellcome Trust and the Health Research Board (Grant Number 203930/B/16/Z), the Health Service Executive National Doctors Training and Planning and the Health and Social Care, Research and Development Division, Northern Ireland. Author SK is funded by the Irish Research Council- Employment Based Postgraduate Program under grant number EBPPG/2021/74.

## Conflict of Interest

Author MO’D is chief scientific officer and equity holder in ONK therapeutics. SK and SP are employees of ONK therapeutics. MG has received research support from ONK therapeutics.

## Publisher’s Note

All claims expressed in this article are solely those of the authors and do not necessarily represent those of their affiliated organizations, or those of the publisher, the editors and the reviewers. Any product that may be evaluated in this article, or claim that may be made by its manufacturer, is not guaranteed or endorsed by the publisher.
